# High-resolution, depth-resolved vascular leakage measurements using contrast-enhanced, correlation-gated optical coherence tomography in mice

**DOI:** 10.1364/BOE.415227

**Published:** 2021-03-02

**Authors:** Conrad W. Merkle, Marco Augustin, Danielle J. Harper, Johanna Gesperger, Antonia Lichtenegger, Pablo Eugui, Gerhard Garhöfer, Martin Glösmann, Bernhard Baumann

**Affiliations:** 1 Center for Medical Physics and Biomedical Engineering, Medical University of Vienna, Vienna, Austria; 2 Division of Neuropathology and Neurochemistry, Department of Neurology, Medical University of Vienna, Vienna, Austria; 3 Department of Clinical Pharmacology, Medical University of Vienna, Vienna, Austria; 4 Core Facility for Research and Technology, University of Veterinary Medicine Vienna, Vienna, Austria

## Abstract

Vascular leakage plays a key role in vision-threatening retinal diseases such as diabetic retinopathy and age-related macular degeneration. Fluorescence angiography is the current gold standard for identification of leaky vasculature *in vivo,* however it lacks depth resolution, providing only 2D images that complicate precise identification and localization of pathological vessels. Optical coherence tomography (OCT) has been widely adopted for clinical ophthalmology due to its high, micron-scale resolution and rapid volumetric scanning capabilities. Nevertheless, OCT cannot currently identify leaky blood vessels. To address this need, we have developed a new method called exogenous contrast-enhanced leakage OCT (ExCEL-OCT) which identifies the diffusion of tracer particles around leaky vasculature following injection of a contrast agent. We apply this method to a mouse model of retinal neovascularization and demonstrate high-resolution 3D vascular leakage measurements *in vivo* for the first time.

## 1. Introduction

Vascular dysfunction plays a critical role in a number of vision-threatening retinal diseases such as diabetic retinopathy (DR) and age-related macular degeneration (AMD), which have been projected to affect a combined 300 million people worldwide by 2020 [1,2], with this number expected to grow to over 500 million by 2040 [1,3]. In wet AMD, new vessels, called neovascularizations (NVs), disrupt the retina, causing lesions and leading to rapid, severe, and irreversible vision loss [4]. These NVs are structurally unstable and can leak their contents into the surrounding tissue. In diabetic retinopathy, focal regions of vascular swelling, called microaneurysms, present at the earliest stages of disease, with NVs developing at later stages [5]. Vascular leakage is therefore an important biomarker for assessing these diseases and developing optimal treatment plans.

The gold standard for identifying vascular leakage in ophthalmology is fluorescence angiography (FA). In FA, a fluorescent contrast agent, most commonly fluorescein or indocyanine green, is injected intravenously, excited at the region of interest by a light source, and the emitted fluorescence is detected by a camera [6]. Typically, the fluorescence signal will be confined to the vasculature, yielding an angiogram. For cases where the blood retinal barrier (BRB) is impaired, tracer may leak out of the vasculature, resulting in a bloom of signal around the affected vessels [7,8]. Unfortunately, conventional FA methods lack depth resolution, making it difficult or impossible to precisely locate the source of the leakage. Optical coherence tomography (OCT), a high resolution optical imaging modality which provides depth-resolved reflectivity information [9], has seen explosive growth in clinical ophthalmology in the last few decades. This growth has largely been driven by OCT’s capacity for rapid volumetric imaging with micron-scale axial resolution, which enables non-invasive observation of the retina’s distinct layers, and by methods such as Doppler OCT and OCT angiography, which quantify the speed and visualize the motion of flowing red blood cells respectively [10]. Despite these strengths, OCT has not been able to visualize vascular leakage in part due to the negligible reflectivity of blood plasma components. Here we propose a new method, exogenous contrast-enhanced leakage OCT (ExCEL-OCT), that uses an OCT contrast agent to measure vascular leakage with high 3D resolution.

Contrast agents have previously been used with OCT to induce changes in the measured signals. Positive OCT contrast agents, which increase the local OCT signal, include lipid particles [11,12], gold nanoparticles and nanorods [13–15], and other highly-scattering nanoparticles [16,17]. Negative OCT contrast agents on the other hand are typically dyes such as indocyanine green [18,19] or methylene blue [20,21] with strong absorption bands in the OCT wavelength range that decrease the local OCT signal. Different contrast agents can be applied for a range of purposes including increasing the intensity [11,13] and Doppler signals [12] within blood vessels [11,12,15] or lymph channels [14,15], correcting intravascular scattering artefacts [22,23], inducing spectral changes [14,15,18], and enabling externally triggerable signal changes [20,21,24]. By tracking the passage of a contrast agent through the vascular system over time, using a method called dynamic-contrast OCT (DyC-OCT), it is also possible to quantify kinetics, blood volume, and blood flow [11,22,25]. In this work, we use the novel ExCEL-OCT methods to visualize the longer-term extravasation of tracer particles from leaky blood vessels in addition to the short-term temporal characteristics of tracer enhancement within the vasculature.

## 2. Methods and materials

### 2.1 Imaging system

A previously described custom-built polarization-sensitive OCT ophthalmoscope for rodent eye imaging was used in this study [26]. This spectral-domain system used a multiplexed superluminescent diode (Superlum, Carrigtwohill, Ireland) with an 840 nm central wavelength and a full width at half maximum bandwidth of 100 nm to provide an axial resolution of 5.1 µm in air (∼3.8 µm in tissue). The eye was illuminated with a power of 2.85 mW, a beam diameter of 0.5 mm, and using circular polarization, and the detected light was split to two spectrometers with line scan cameras (Basler AG, Ahrensburg, Germany) which measured perpendicular polarization states; however for the new ExCEL-OCT methods described in this work, only the co-polarized OCT channel was used, similar to conventional OCT. Sensitivity was measured at 96 dB when running these cameras at an 83 kHz A-line rate and using 3072 of the available 4096 camera pixels per camera to increase acquisition speed. Relative to the previously published system design [26], this ophthalmoscope now features a sample arm telescope to correct focal length mismatches in the rodent eye and reduce the beam diameter at the pupil as shown in a previous study [27].

### 2.2 Contrast agent

Intralipid 20% (Fresenius Kabi Austria GmbH, Graz, Austria) was used as a positive, highly scattering, OCT contrast agent. Intralipid 20% is an emulsion of lipid particles that has on-label uses as an intravenously delivered nutritional supplement for use in humans. These lipid particles range in size from a few tens of nanometers to over 600 nm in diameter with a mean of around 214 nm [28]. Previous studies have demonstrated that Intralipid can also be used as a biocompatible plasma tracer [11] and can be used to improve the OCT intensity, angiography, and Doppler signals [11,12]. It has further been shown that Intralipid 20% preferentially enhances the intravascular OCT signal in vessels which are parallel to the beam axis, thus improving their visibility [23,29]. Intralipid 20% has been chosen here for its ease of use, translational potential, and lipid makeup which is expected to be beneficial for retinal leakage imaging. The intact blood-retinal barrier is naturally susceptible to lipid transport [30], and this effect is predicted to be enhanced around leaky neovascularizations with an impaired blood-retinal barrier.

### 2.3 Animal model and procedures

A very low density lipoprotein receptor (-/-) (VLDLR^-/-^) knockout mouse model (n = 10, B6;129S7-Vldlr^tm1Her^/J, The Jackson Laboratory, Bar Harbor, USA) was used to study leaky vasculature in the retina *in vivo*. This model is an established model of retinal neovascularization [31], which reliably produces leaky vasculature starting around 3 weeks of age. Unlike other models of neovascularization, which originate in the choroid, the VLDLR model follows a progression similar to retinal angiomatous proliferation [32], where new vessels grow from the outer plexiform layer of the inner retina toward the subretinal space, eventually disrupting the retinal pigment epithelium and forming retinal-choroidal anastomoses. As a control, wild type (WT) mice with a B6SJL background (n = 6, The Jackson Laboratory, Bar Harbor, USA) were used. Mice were anesthetized with either isoflurane or ketamine/xylazine. Isoflurane was vaporized using an induction concentration of 4% for 4 minutes before reducing to 2% for imaging. Isoflurane was mixed with oxygen and delivered at a rate of 1.5-2 L/min. Excess or exhaled isoflurane was scavenged with a vacuum. A small number of VLDLR mice (n = 3) did not remain sufficiently anesthetized under the reduced 2% concentration of isoflurane anesthesia and were instead given a mixture of ketamine and xylazine. These mice received a 10 mL/kg body weight intraperitoneal injection of ketamine/xylazine, mixed using 0.15 mL xylazine (Rompun, 20 mg/mL, Bayer AG, Leverkusen, Germany), 0.5 mL ketamine (Ketasol, 100 mg/mL, aniMedica GmbH, Frankfurt, Germany), and 4.35 mL isotonic saline, resulting in a dose of 100 mg/kg ketamine and 6 mg/kg xylazine.

Mice were placed on a heating pad in a custom multi-axis mount for head stabilization and precise orientation in front of the OCT ophthalmoscope. A drop of Tropicamide (5 mg/mL, Agepha Pharma s.r.o., Senec, Slovakia) was applied to each eye to dilate the pupil and artificial tear drops (Oculotect, ALCON Pharma GmbH, Freiburg im Breisgau, Germany) were applied throughout the imaging experiment to keep the eyes moisturized. A 3 mL/kg body weight bolus injection of Intralipid 20% was delivered via the tail vein to produce OCT contrast for 3D leakage imaging. All animal protocols have been approved by the ethics committee of the Medical University of Vienna and the Austrian Ministry of Education, Science, and Research (BMBWF/66.009/0272-V/3b/2019).

### 2.4 Scan protocols

**Fig. 1.**
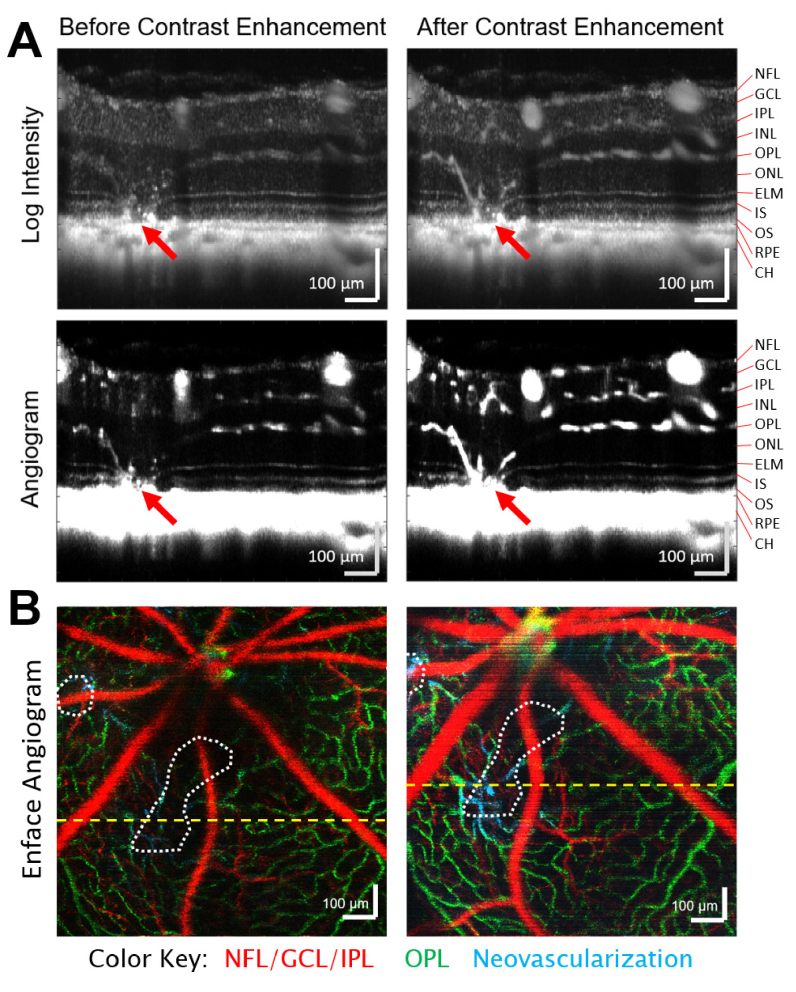
Volumetric OCT scans provide depth-resolved structural and angiographic information, which can be enhanced with an injectable tracer. A) Intravenous injection of Intralipid 20% as an OCT contrast agent dramatically improves the OCT intensity and angiogram signals within both healthy vasculature and neovascularizations (red arrows). Log intensity and angiogram images are shown both before and during peak enhancement of the injection with 100 averages from a single dynamic-contrast OCT scan. These pairs of intensity and angiogram images are shown with the same dynamic range. B) Color-coded enface angiograms from pre- and post-injection volumetric OCT angiography scans are segmented by depth and maximum intensity projected to highlight the vasculature of the superficial (red), and deeper (green) vascular layers. Neovascularizations that inhabit typically avascular layers are shown in cyan and the bounds of lesions are denoted by dotted white lines. The locations of the above cross-sectional scans are given by the yellow dashed lines. NFL – Nerve Fiber Layer, GCL – Ganglion Cell Layer, IPL – Inner Plexiform Layer, INL – Inner Nuclear Layer, OPL – Outer Plexiform Layer, ONL – Outer Nuclear Layer, ELM – External Limiting Membrane, IS – Inner Segments, OS – Outer Segments, RPE – Retinal Pigment Epithelium, CH – Choroid.

A traditional 3D angiography scan protocol covering a field-of-view of 1 mm × 1 mm centered on the optic nerve head was used to generate both angiogram and leakage maps. This protocol used a scan density of 500 A-lines per B-scan with 5 B-scan repeats at each of 400 slow-axis positions for a post-Fourier transform data set with a size of 1536 × 500 × 400 × 5 pixels (z,x,y,t). These angiogram scans were performed multiple times per eye both before and after injection of the OCT contrast agent to track the leakage of tracer particles. A DyC-OCT scan protocol, which sampled the same B-scan cross-section repeatedly, was also used to scan along a neovascularization during the first passage of the contrast agent [Fig. 1(A)]. The size of the DyC-OCT scan was similarly 1536 × 500 × 2000 pixels (z,x,t).

### 2.5 Data processing

#### 2.5.1 Motion correction and flattening

Relative to imaging at other body locations, it is difficult to stabilize the eye through physical means to reduce motion during imaging. For this reason, post-processing methods were used to compensate for motion and flatten the retina. Here, the strong intensity of the retinal pigment epithelium in the cross-polarized OCT channel was used to align frames and flatten the retina as described in our previous work [33,34]. All other processing steps were performed solely on the co-polarized OCT channel.

#### 2.5.2 Leakage processing

**Fig. 2.**
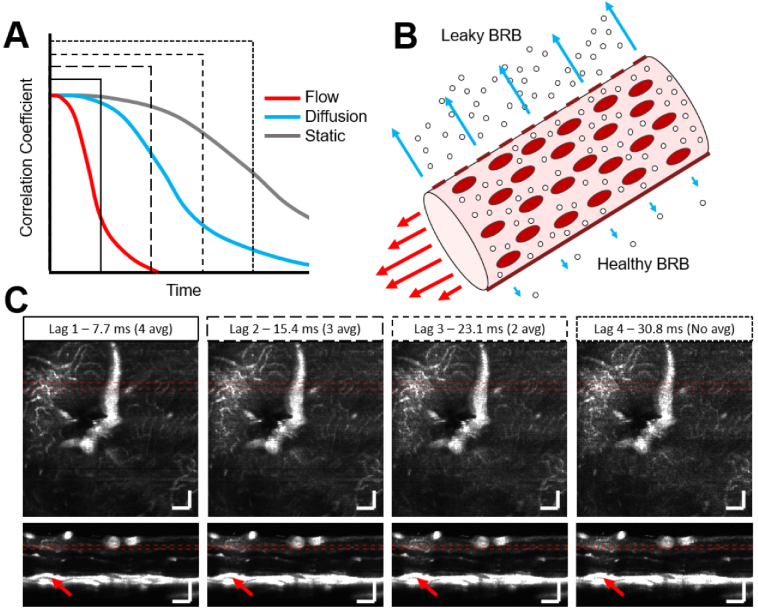
Selecting a longer angiogram interscan time increases the sensitivity to signals that decorrelate more slowly. A) In most cases, intravascular flow signals (red) will decorrelate most quickly due to the high speed of tracer particles and red blood cells through the field of view. Diffusion (cyan) is typically slower than flow and signals will therefore decorrelate more slowly. OCT signals in static tissue (grey) decorrelate the slowest. The four line styles represent four different interscan, or lag, times used in ExCEL-OCT processing and shown in C) below. B) Impairment of the blood retinal barrier (BRB) is expected to allow more tracer particles to extravasate relative to a healthy BRB. C) As interscan time is increased, the resulting angiograms become more sensitive to slower decorrelation rates, as can be seen above a retinal lesion (red arrow). This region can be seen in more detail in Fig. S1. Mean intensity projections through a leakage hotspot are shown over 40 µm laterally or 20 µm axially as denoted by the dotted red lines in the enface and B-scan images. Scale bars are 100 µm and all figures are shown with the same dynamic range.

In order to visualize leakage, we developed novel data processing methods to highlight the scattering signal from the extravasated Intralipid particles. To discriminate between the static tissue, blood flow, and the desired leakage signals, we have implemented a selective decorrelation gate. Decorrelation describes how quickly the signal within a given voxel is changing as a function of time [Fig. 2(A)]. The key idea is that OCT signals in static tissue will decorrelate slowly because the tissue is not moving. The signal within vessels, on the other hand, will decorrelate rapidly due to the relatively high speed of the red blood cells and Intralipid particles passing through the voxel. Finally, the extravasated Intralipid particles, which are driven by slower diffusion processes rather than blood flow, will have a decorrelation rate that falls somewhere between the other two. While lipid particles may cross the blood retinal barrier (BRB) in healthy vessels, it is expected that much stronger extravasation and diffusion signal will be observed around leaky vessels [Fig. 2(B)]. To separate the signal contributions from different decorrelation rates, we use a form of variable interscan time analysis (VISTA) [35].

In traditional OCT angiography methods, repeated B-scans are acquired at the same position and the change in signal between adjacent B-scans is evaluated, typically revealing intravascular flow. VISTA methods evaluate the change in signal between both adjacent and non-adjacent B-scans to coarsely sample decorrelation. VISTA has previously been used at very high frame rates in order to perform intravascular velocimetry [36]. Here we move away from short interscan times and instead use longer interscan times to highlight diffusing tracer particles. In this case, a complex-subtraction angiography method, similar to traditional angiography methods [37], is used with 5 B-scan repeats, yielding 4 possible interscan, or lag, times [Fig. 2(C)]. With a B-scan frame rate of 130 Hz and 1–4 lag frames, we cover a range from 7.7–30.8 ms in 7.7 ms increments. Increasing the number of lag frames also decreases the number of valid permutations that can be used for angiogram averaging. As interscan time increases, the heightened sensitivity to slower decorrelation rates can be observed [Fig. 2(C)]. The equation for the average bulk phase-corrected angiogram with a given lag (L) is shown below where S is the complex OCT signal, φ_corr_ denotes the bulk axial phase correction described by Lee et al. [38] and defined below, Δt is the interscan time of the OCT system, N is the number of B-scan repeats at each slow axis position, and * is the complex conjugate.

(1) $$A_{L}(z,x,y)=\frac{1}{N-L}\sum_{t=0}^{(N-L)\Delta t}|S(z,x,y,t+L\Delta t)-S(z,x,y,t)exp\{i\varphi_{corr}(x,y,t,L)\}{|}^{2}$$

(2) $$\varphi_{corr}(x,y,t,L)=arg\{\int S(z,x,y,t+L\Delta t)S^{*}(z,x,y,t)\;dz\}$$

**Fig. 3.**
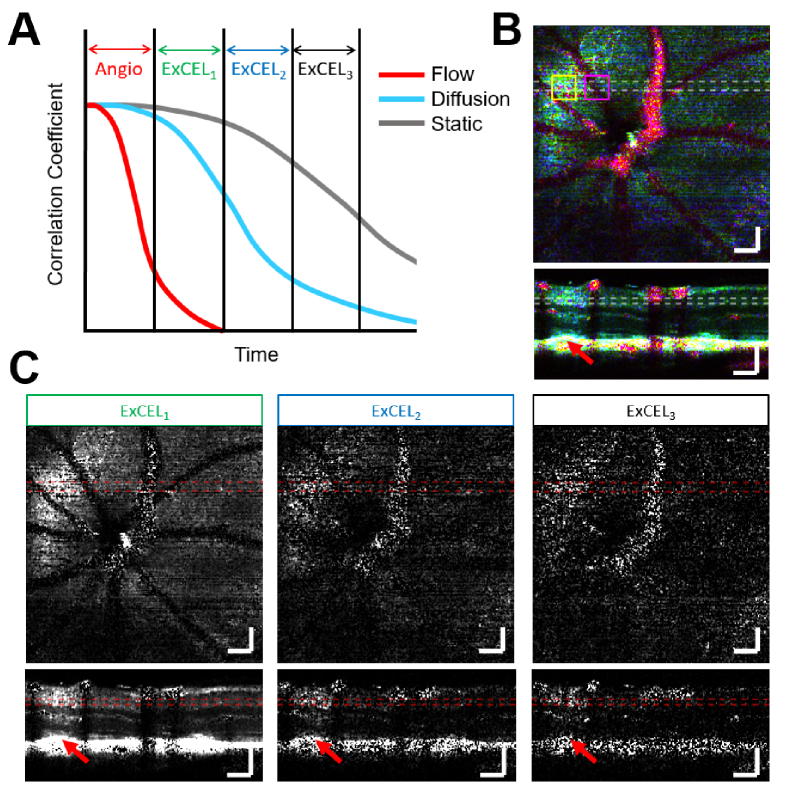
The ExCEL signals described in Eq. (3) use decorrelation gates to exclude intravascular flow and highlight diffusion-driven extravascular leakage. A) ExCEL signals with later decorrelation gates visualize slower diffusion. B) A false color leakage map can be generated by overlaying the traditional angiogram signal (red) with the ExCEL_1_ (green) and ExCEL_2_ (blue) signals. C) ExCEL components with different decorrelation gates are shown individually. A bloom of leakage signal is visible above the retinal lesion marked with a red arrow. This region can be seen in more detail in Fig. S2. Images are shown as mean intensity projections over 40 µm laterally or 20 µm axially as marked by the dashed lines in the enface and B-scan images. These images are shown at the same location as Fig. 2(C). ExCEL images are all shown with the same dynamic range and scale bars are 100 µm. Yellow and magenta boxes mark ROIs containing strong leakage and vascular signals which are further analyzed in Fig. 5 and Fig. 6 respectively.

Looking at Fig. 2(C), with the traditional lag 1 angiogram, the intravascular signal is clear, however diffusion is not well visualized. At lag 2-4, a cloud of signal becomes progressively more apparent in the plexiform layers above a retinal lesion. This can be seen in more detail in Fig. S1. While longer lag times appear to be more sensitive to diffusion, these images still also include information from intravascular flow. To more specifically highlight leakage of different diffusion rates and remove the intravascular signal, angiogram signals of different lag times can be subtracted from each other, generating a decorrelation-gated signal [Fig. 3]. The resulting exogenous contrast-enhanced leakage signal is termed ExCEL for short with a subscript designating the number of lags beyond the traditional angiogram signal.

(3) $$ExCEL_{L-1}(z,x,y)=A_{L}(z,x,y)-A_{L-1}(z,x,y)$$

Because the angiogram data sets of different lags are perfectly coregistered, this subtraction cleanly removes most of the intravascular contributions from the leakage signal [Fig. 3]. Because ExCEL_3_ was comparatively noisy due to the lack of averaging, we focused our attention on ExCEL_1_ and ExCEL_2_ in this work. By coding the information from the traditional angiogram and the first two ExCEL channels into red, green, and blue colors respectively, we can combine all of this information into a single image for evaluation purposes [Fig. 3(B)]. A zoomed-in view of the leakage region for each channel and the composite color image can be found in Fig. S2.

#### 2.5.3 Depth profiles

In one VLDLR animal, depth profiles for the angiogram, ExCEL_1_, and ExCEL_2­_ signals were produced at multiple time points before (-5 and -2.5 minutes) and after (+0.5, +2, and +3.5 minutes) tracer injection to observe the time-dependent change in signals. First the angiogram, ExCEL_1_, and ExCEL_2­_ signals were calculated as described above and smoothed by convolving with a 3 × 3 × 3 kernel of ones divided by 27 to reduce noise. The background noise level, measured in a region of the vitreous above the retina containing no scattering signal and averaged over the field of view, was then subtracted from each signal. ROIs with lateral dimensions of 100 µm × 100 µm were selected in a region of high leakage signal post-injection, marked by the yellow box in Fig. 3(B), and a neighboring vascular reference region, marked by the magenta box in Fig. 3(B). These regions were selected to investigate both extravascular and intravascular contributions of the tracer. Analysis of a third region outside of both leakage and vascular ROIs can be seen in Fig. S3. These regions were chosen close together to minimize differences in focusing effects and use the same range of slow-axis positions to minimize the effects of motion. ROIs were first manually coregistered laterally, and the signals were averaged along the fast and slow axes to generate individual depth profiles for each time point. While manual coregistration has an expected error of a few microns based on human error, by averaging the signal over 100 × 100 µm ROIs, we ensure good overlap of these regions, which yields reliable depth profiles for each time point. Using a larger ROI also makes the measurements more sensitive to accumulation effects in the tissue, which are expected to be low intensity and dispersed, as opposed to intravascular changes caused by tracer injection, which are higher intensity and contained within the vessels. Next, automatic alignment of the axial position was achieved using a cross-correlation method. To compensate for the general drop in intensity over time due to evaporation of the tear-film layer or development of cataracts, each signal was normalized to the maximum lag 3 angiogram signal for that ROI and time point. The lag 3 angiogram signal was used for this normalization as it was less noisy than the lag 4 angiogram, but still provided signals that are predominantly decorrelated for all time points even for slight variations in motion between volumes. The relative change in signals was also measured by dividing by the mean baseline depth-dependent signal before injection.

#### 2.5.4 Automated processing

**Fig. 4.**
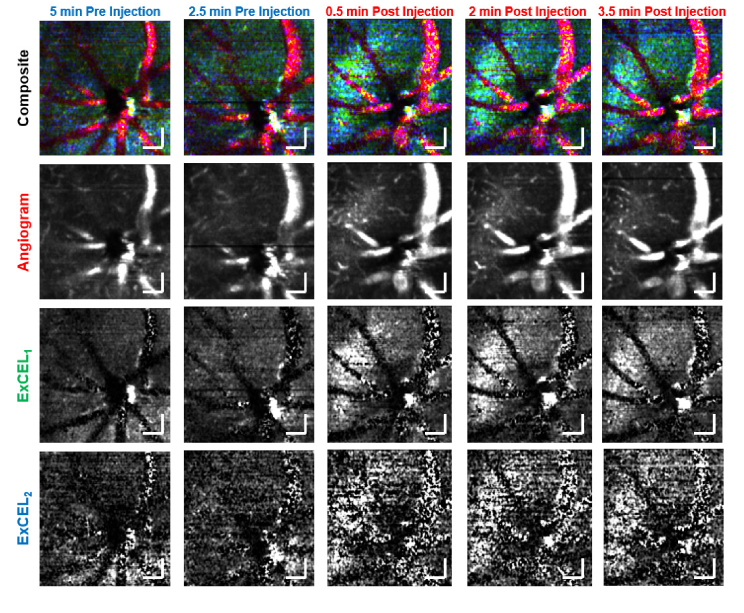
Individual frames from the automated movie processing used for blind leakage grading. These images form a time course of the leakage signal at the same position for 2 time points before and 3 time points after injection of the Intralipid tracer. A clear increase in leakage signal (cyan) is observed following injection. Individual color channels shown are below for the angiogram (red), ExCEL_1_ (green), and ExCEL_2_ (blue) signals. Scale bars are 100 µm.

Flythrough videos of the volumetric leakage data were generated in a fully automated fashion with a goal of accommodating data sets with highly variable signal to noise ratios (SNR). For each data set, angiogram and ExCEL signals were calculated as described by Eq. (3). Each signal was then convolved with a 5 × 5 × 5 matrix of ones divided by 125 to smooth out speckle noise. The mean background noise was measured in the vitreous region above the retinal surface and subtracted from each volume. Each frame of the movie was then generated as the enface mean intensity projection over a 10 µm depth range with each frame shifting by 1 pixel (∼2 µm) in depth. The angiogram signal was coded into the color red, ExCEL_1_ was coded into green, and ExCEL_2_ was coded into blue as shown in Fig. 3(B) and Fig. 4. The dynamic range for each signal in each movie frame was set from 0 to 5 times the mean signal in the frame in order to accommodate the large variability of signal levels in different animals, and at different time points or depth positions. In this way, signals with average intensity appeared dim, and regional increases in signal would appear bright for the respective color channels. An example video with a side-by-side comparison of pre- and post-tracer injection flythroughs from the VLDLR mouse in Fig. 4 is available in the Supplement 1 (Visualization 1).

#### 2.5.5 Blind grading

Leakage mapping was applied in an automated fashion using the above methods to 58 OCT angiography volumes from VLDLR mice before (n = 20) and after (n = 38) tracer injection. An additional 25 volumes from WT mice before (n = 21) and after (n = 4) tracer injection were processed to act as a control and establish the baseline false positive error rate caused by misinterpretation of artefacts in the ExCEL signals. An example of a common ring-shaped artefact along with a quantitative analysis of the ExCEL signals in this region in a WT mouse before and after injection can be seen in Fig. S4. Each data set was stored as a depth-wise flythrough MATLAB movie. Blind grading was performed by 4 different graders to assess the presence or absence of leakage in each data set. Graders were trained on a small subset (∼6%) of the data to identify vascular leakage while avoiding artefacts caused by surface reflections or other structural changes. Graders performed binary scoring for each data set with a 0 indicating no leakage in the volume and a 1 indicating the presence of leakage. A custom MATLAB program was used to randomize and present each leakage movie in sequence with each grader receiving a different order of files. Graders had full control to play through the data, rewind, and step through individual frames. All 83 volumes were graded in a single sitting, typically requiring approximately one hour per grader. Statistical testing of this categorical data used Fisher’s exact test.

## 3. Results

### 3.1 Enhanced visibility of neovascularizations

Figure 1 demonstrates that Intralipid dramatically improved the visibility of neovascularizations growing into a retinal lesion. While some neovascularizations could be seen before the injection, significant increases in OCT intensity and angiogram signals were observed within the vasculature following the contrast agent injection.

### 3.2 Leakage time course

**Fig. 5.**
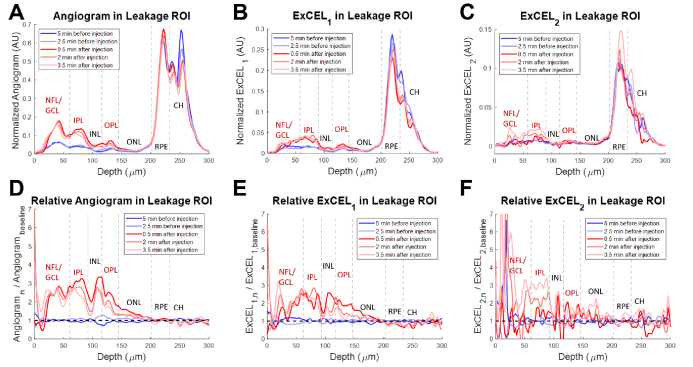
Depth profiles from a VLDLR mouse eye for the traditional angiogram (A), ExCEL_1_ (B), and ExCEL_2_ (C) signals averaged over the 100 µm × 100 µm leakage region shown in Fig. 3(B) (yellow box). Relative changes in signals (D-F) were also measured by dividing by the mean baseline profile. Blue profiles were acquired before Intralipid injection and red profiles were acquired after injection. NFL – Nerve Fiber Layer, GCL – Ganglion Cell Layer, IPL – Inner Plexiform Layer, INL – Inner Nuclear Layer, OPL – Outer Plexiform Layer, ONL – Outer Nuclear Layer, RPE – Retinal Pigment Epithelium, CH – choroid. Vascular layers of the inner retina are labeled in red.

**Fig. 6.**
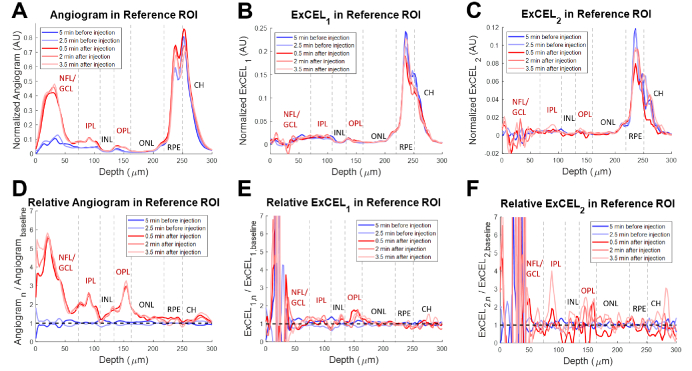
Depth profiles from a VLDLR mouse eye for the traditional angiogram (A), ExCEL_1_ (B), and ExCEL_2_ (C) signals averaged over the 100 µm × 100 µm vascular reference region shown in Fig. 3(B) (magenta box). Relative changes in signals (D-F) were also measured by dividing by the mean baseline profile. Blue profiles were acquired before Intralipid injection and red profiles were acquired after injection. NFL – Nerve Fiber Layer, GCL – Ganglion Cell Layer, IPL – Inner Plexiform Layer, INL – Inner Nuclear Layer, OPL – Outer Plexiform Layer, ONL – Outer Nuclear Layer, RPE – Retinal Pigment Epithelium, CH – choroid. Vascular layers of the inner retina are labeled in red.

Using the fully-automated leakage movie processing, there was a clear increase in ExCEL signals (green and blue) in extravascular regions following injection of tracer as shown in Fig. 4. This change was further investigated as a function of depth in a leakage ROI [Fig. 5] and a vascular reference ROI [Fig. 6].

#### 3.2.1 Depth profiles in the leakage ROI

While the absolute angiogram signal demonstrated three peaks in the vascular layers of the inner retina [Fig. 5(A), labeled in red], i.e. surface vasculature from the nerve fiber and ganglion cell layers (NFL and GCL) and the capillaries of the inner and outer plexiform layers (IPL and OPL), the relative change in angiogram signal due to the tracer injection [Fig. 5(D)] demonstrated a rounded appearance stretching into the typically avascular inner and outer nuclear layers (INL and ONL). A strong increase followed by a slight decay in angiogram signal was noted over time following injection. The ExCEL_1_ profile [Fig. 5(B)] appeared more flat than the angiogram profile with the most prominent increase in signal occurring in the IPL following injection. Nevertheless, the relative ExCEL_1_ profile [Fig. 5(E)] demonstrated a similar rounded appearance to the relative angiogram profile. Additionally, a decay in ExCEL_1_ signal in the INL, OPL, and ONL was observed over time. The ExCEL_2_ profile [Fig. 5(C)] was similar in appearance to the ExCEL_1_ profile, however the relative change in signal [Fig. 5(F)] demonstrated little to no change at 30 seconds following injection, with increasing signal in the IPL at later time points. Noise was highest in the ExCEL_2_ signal with significant fluctuations in the relative profile at the retinal surface due to low signal. The enhancement factor of both ExCEL signals in the leakage ROI peaked around 2-3 following contrast agent injection. The baseline profiles showed good agreement with each other, and no large changes were observed in the retinal pigment epithelium or choroid following injection.

#### 3.2.2 Depth profiles in the reference ROI

Angiogram signals in the absolute profiles for the vascular reference ROI were primarily isolated to the vascular layers of the inner retina [Fig. 6(A), labeled in red]. A large relative increase in angiogram signal was similarly observed predominantly in the inner retinal vascular layers following the injection of tracer [Fig. 6(D), labeled in red]. This increase confirms that tracer particles are present in the vascular system at all time points following injection. Absolute profiles of ExCEL signals [Fig. 6(B)–6(C)] showed low, flat signals in the inner retina, and the relative change profiles [Fig. 6(E)–6(F)] both demonstrated no apparent change in ExCEL signals following injection aside from noise. These ExCEL profiles were consistent with additional reference profiles obtained from the same VLDLR mouse [Fig. S3] and a WT mouse [Fig. S4]. The baseline profiles showed good agreement with each other, and no large changes were observed in the retinal pigment epithelium or choroid following injection.

### 3.3 Leakage grading

**Fig. 7.**
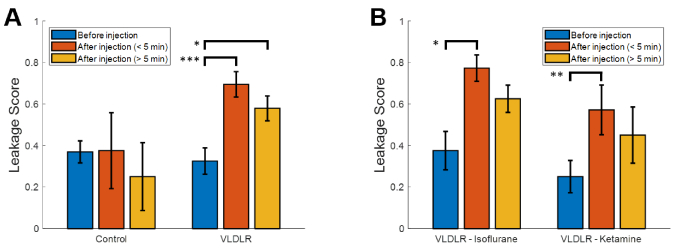
Blind grading of leakage data sets by 4 graders. A) Average leakage scores in control and VLDLR mice before and after injection of Intralipid. B) Leakage scores for VLDLR mice sorted by anesthesia type. Error bars show the standard error which is defined as the standard deviation divided by the square root of the number of samples. Asterisks indicate the level of statistical significance using the Fisher exact test (* p < 0.05, ** p < 0.005, and *** p < 0.0005).

Blind grading of VLDLR and control data sets before and after injection of Intralipid revealed clear and significant (p < 0.05) increases in the average leakage grade following injection for VLDLR mice [Fig. 7(A)]. This increase was particularly apparent for data sets acquired within the first 5 minutes following the injection (p < 0.0005). Similar trends were also observed by individual graders, of whom, 3 of 4 found a statistically significant (p < 0.05) increase in leakage grade between baseline and the first 5 minutes following injection for VLDLR mice. Examination of leakage scores in the VLDLR mice under different anesthesia types [Fig. 7(B)] demonstrated lower average scores under ketamine anesthesia, however this difference was not statistically significant. Leakage scores under both types of anesthesia demonstrated statistically significant increases (isoflurane, p < 0.05 and ketamine, p < 0.005) following injection in VLDLR mice. Pre-injection VLDLR leakage scores were not statistically significantly different from pre-injection WT scores (p = 0.75). Post-injection WT leakage scores were similar to pre-injections scores, however sample numbers were too low to draw statistically significant conclusions.

## 4. Discussion

### 4.1 Performance of Intralipid as a leakage contrast agent

Intralipid is an ideal contrast agent for retinal leakage imaging due to the blood retinal barrier’s natural susceptibility to lipid transport [30]. The results presented above demonstrated a clear increase in ExCEL-OCT signal [Fig. 4] and leakage grade [Fig. 7] for VLDLR mice following injection. This increase in ExCEL-OCT was also visible in the quantitative comparison of depth profiles located above a retinal lesion before and after injection [Fig. 5]. ExCEL signals were visible as a bloom of signal with a circular or spherical appearance and predominantly excluded intravascular signal. The leakage signal further appeared to remain fairly localized given the lack of ExCEL signal in the nearby reference regions [Fig. 6 and Fig. S3]. We similarly did not observe changes in ExCEL signals following injection in a WT mouse [Fig. S4]. While it is possible that the Intralipid particles may cross the healthy blood-retinal barrier, we did not observe this phenomenon in this study. It is expected that this type of leakage would present uniformly around vessels throughout the eye. The automated processing used to generate movies for leakage grading would potentially suppress this effect as it was designed to highlight focal points of leakage signal associated with our disease model. In our quantitative comparison of WT ExCEL signals [Fig. S4], no change was observed, suggesting that the concentration of extravasated particles around healthy vessels may be too low to detect using OCT at this wavelength; however a follow-up study focused on WT mice would be required to confirm this finding.

The leakage grading suggests that the extravasated tracer is removed from the system on the order of minutes, which is in line with the intravascular half-life for Intralipid of approximately 9 minutes previously reported for rats [39]. However, we cannot rule out that the reduced leakage grade at later time points is instead caused by a progressive drop in diffusion speed and signal as the tracer thins out or from reduced signal quality due to drying of the tear film or development of cataracts over time. Other contrast agents such as customized nanoparticles may be able to provide higher sensitivity for leakage measurements, but these have less translational potential as they are not currently approved for human use. Intralipid 20% and other similar lipid emulsions, on the other hand, are approved for human use both in the United States and in Europe.

### 4.2 Performance of Intralipid for angiography of neovascularizations

As reported previously, the injection of Intralipid particles increases the intravascular intensity and angiogram signals as measured with OCT [11]. It has also been demonstrated that the vessels that run parallel to the beam axis exhibit the largest increases in scattering [29]. Due to the growth of NVs between the choroid and outer plexiform layer in a significantly vertical orientation, the visibility of NVs increased substantially following Intralipid injection and the boundaries of these vessels became much more apparent [Fig. 1]. The rounded shape of the traditional angiogram depth profile [Fig. 5(D)] even in layers that should contain very little vasculature indicates that the angiogram signal does pick up some degree of leakage signal. Therefore, a better separation of angiogram and leakage signals may be achieved by using a shorter interframe time for higher temporal resolution.

### 4.3 Comparison of depth profiles

The signal profiles quantified in Fig. 5 and Fig. 6 demonstrate clear differences between the leakage and reference ROIs. While both angiogram profiles demonstrate an increase in signal following injection, the change in the reference ROI was primarily confined to the vascular layers with three clear peaks. The change in the leakage ROI by comparison was much broader, spreading into the INL and ONL, indicating contributions from a bloom of contrast agent that had leaked into the surrounding tissue. In addition to the angiogram signal, the ExCEL_1_ signal demonstrated a similar broad increase in the leakage ROI, but no apparent change in the reference ROI. The ExCEL_2_ signal demonstrated a narrower increase following injection in the leakage ROI that grew in strength over time after a delay, but no change in the reference ROI. This also coincided with a slight decrease in signal over time in the angiogram and ExCEL_1_ signals, particularly in the INL, OPL, and ONL. The increase in ExCEL_2_ signal and the decreases in the angiogram and ExCEL_1_ signals over time may be related to slower diffusion speed as the tracer particles move further from the leaky vasculature and are driven by weaker concentration gradients. The stark differences in signal changes within the leakage and reference ROIs following injection point towards selective identification of leakage using the ExCEL signals. It is unlikely that these changes in signals would be caused by differences in focus or motion artefacts as they were acquired from neighboring regions selected from the same set of slow-axis positions, therefore any changes that would affect one ROI, should affect the other ROI similarly. The findings in the reference region ExCEL profiles [Fig. 6] were consistent with additional reference profiles obtained from the same VLDLR mouse [Fig. S3] and a WT mouse [Fig. S4].

### 4.4 Influence of anesthesia on leakage grading

While not the focus of this paper, lower leakage grades were observed in VLDLR mice under ketamine compared to isoflurane anesthesia. Although this was not found to be statistically significant, isoflurane is a vasodilator and may conceivably increase permeability of Intralipid across compromised blood retinal barriers similar to observations in the blood brain barrier following traumatic brain injury [40]. Nevertheless, fewer ketamine data sets were obtained and the mice were on average younger than those who received isoflurane, so a more rigorous study that includes monitoring of perfusion pressure should be conducted in the future to evaluate this observation.

### 4.5 Evaluation of performance

ExCEL-OCT provides visualization of leakage following injection of a tracer, however there are certain effects to consider when interpreting these images. While sensitive to slower, diffusive motion, the presented method is qualitative and is not able to exclusively distinguish diffusion from slow intravascular flow or bulk motion of static tissue. In particular, a false-positive leakage signal from the motion of highly-scattering structures is present similar to OCT angiography. This effect is expected to be less pronounced as OCT systems become faster and motion compensation improves, making leakage measurements more stable. Faster scanning with additional averages would also enable shorter interscan times and a higher temporal resolution for ExCEL measurements.

Despite some contamination of the leakage signal due to motion, we present strong evidence that the leakage signal is specific to tracer leakage rather than other artefactual effects. By tracking the same eye regions over time both before and after injection, we show a selective increase in ExCEL leakage signals above a retinal lesion relative to a neighboring reference region which demonstrated no changes. The angiogram depth profile also demonstrated a unique, broad shape in the leakage ROI following injection, which we argue was caused by rapidly moving contrast particles in the leakage bloom. Furthermore, the location of the leakage signal proved to be highly reproducible across time points despite some movement of the mouse eye between scans. In the randomized grading, there was a clear, statistically significant increase in the leakage score for VLDLR mice after injection. Additionally, quantitative analysis of a region in a WT mouse containing ExCEL signal artefacts [Fig. S4] did not demonstrate an apparent change in signal over time.

It was noted that false positive leakage grades occurred in approximately one third of the control and pre-injection VLDLR data sets. The leakage video processing used for the grading was designed to be fully automated to avoid any biases in the video production, however in the future this software can be further refined by adjusting the dynamic range and suppressing artefacts to reduce the false positive rate. This rate may also decrease as graders obtain more experience viewing ExCEL data.

In terms of additional processing demand, ExCEL OCT is comparable to existing angiography methods and is therefore not expected to generate substantial additional processing burden. While some signal from the leakage bloom could be found in the angiogram signal in the depth profile comparison, the ExCEL signals were more specific to the leakage ROI and did not change in the reference ROI. The ExCEL signals are therefore separable from the intravascular signals, giving ExCEL-OCT spatially and temporally coregistered and separable structural, angiographic, and leakage information with high volumetric resolution from a single scan. In the future, different scan patterns with higher frame rates and more averages should be investigated optimize the ExCEL metrics and further separate the angiogram and ExCEL signals.

### 4.6 Comparison with state-of-the-art

While the new methods described here for 3D visualization of lipid leakage using contrast-enhanced OCT have clear benefits over traditional fluorescence angiography and OCT methods, it makes sense to also compare these methods with other state-of-the-art imaging techniques.

Although traditional fluorescence angiography provides only flat 2D images, recent exciting work using oblique scanning laser ophthalmoscopy (oSLO) fluorescence angiography seeks to introduce depth-resolution through a novel scanning and detection geometry [41,42]. This approach has the advantage of access to traditional fluorescence contrast agents and potentially higher detection sensitivity to individual tracer particles. Nevertheless, ExCEL-OCT is able to achieve higher axial resolution which also does not degrade outside the focal spot. Compared to the ∼25 µm axial resolution presented for oSLO [41], our measurements were performed with ∼4 µm axial resolution. The oblique scanning geometry for oSLO also introduces unique artefacts and complicates the image reconstruction and system design, while ExCEL-OCT is more straightforward, using standard OCT systems and angiography protocols and similar reconstruction, making it easier to implement clinically. Finally, to acquire structural data, this oSLO system [41] requires simultaneous acquisition with OCT, further increasing its complexity. ExCEL-OCT on the other hand provides spatially and temporally coregistered structural, angiographic, and leakage information from the same data through post-processing.

Ophthalmic two photon microscopy (TPM) has also been investigated for introducing depth resolution to FA at high resolution. Ophthalmic TPM has been demonstrated both in animal models [43,44] and humans [45] *in vivo*, however results in humans have been limited to the anterior chamber of the eye thus far. This is due in part to the high energy density required to elicit two photon fluorescence, the relatively low exposure limits for the retina, and the difficult imaging geometry associated with retinal imaging in the posterior chamber. Similar to oSLO, TPM also requires a secondary modality to provide corresponding structural information.

To our knowledge, this is first time that OCT has been used to directly visualize the leakage of vascular contents into the surrounding tissue *in vivo*; however indirect visualization of edema formation has previously been demonstrated using a technique referred to as OCT-Leakage [46]. This method examines the reduction in OCT signal associated with significant edema formation and does not directly visualize the process of leakage, but rather the large-scale structural disruption and hyposcattering regions associated with the resulting edema. ExCEL-OCT may therefore be more sensitive to the early stages of vascular leakage than OCT-Leakage. Another promising method uses spectral processing with visible light OCT to visualize red blood cells [47]. While partially developed for angiography, this method has also visualized diffusion of red blood cells in an agarose phantom. Whether vascular leakage could also be measured *in vivo* using this technique remains to be seen.

One final technique of interest is magnetic resonance imaging (MRI), which has a long history of imaging leakage and diffusion in the brain [48,49]. Ophthalmic applications of MRI have also been investigated for non-invasive whole eye imaging [50]. While high isotropic or near-isotropic spatial resolution (42-84 µm) has been reported in the rat eye using a custom 11.7 T system [51], the acquisition time was comparatively long (5-15 minutes). The long scan times associated with high spatial resolution MRI make eye imaging difficult due to motion, which cannot be fully eliminated in *in vivo* measurements. In this particular case, a strong paralytic typically used for terminal animal studies, pancuronium bromide, was used, and the conjunctiva of the eye was sutured directly to the radiofrequency coil to reduce motion [51]. While temporal resolution in MRI can be improved, this trades off with spatial resolution, thereby reducing the depth-resolution of the measurements.

### 4.7 Future work

The work presented here provides a starting point for future 3D leakage studies. An important next step will be to rigorously characterize the leakage signal as a function of time post-injection. Much like fluorescence angiography acquires images at specific time points to visualize arterial phase, venous phase, etc., ExCEL-OCT could be improved by identifying the optimal time points for highlighting leakage. The leakage blooms that we observed here were recorded at the earliest, 30 seconds following tracer injection, and by this time, diffusion of the particles was already substantial. The under 30 second range following injection could therefore provide better spatial identification of leakage sites. We qualitatively observed that leakage signals were predominantly identified above some, but not all retinal lesions. Further analysis of leakage proximity to lesion sites, not only laterally but also axially, is warranted.

Higher sensitivity to Intralipid leakage may be possible using a particle-size filtered Intralipid injection that maximizes reflectivity and diffusivity across the BRB. Smaller particles are expected to cross this barrier easier and diffuse faster, however the scattering cross-section may also reduce OCT signal. Characterization of ExCEL signals as a function of particle size could therefore lead to a more sensitive tracer by balancing these tradeoffs; however the stability of particle-size filtered emulsions would also have to be evaluated to ensure larger particles do not reform following filtering.

There is additionally significant potential to further refine and improve the automated post-processing described here to enable more automatic and quantitative, rather than human-dependent, grading and identification of leakage. A comparison of ExCEL signal quality using different angiography algorithms such as complex subtraction, phase variance, or decorrelation-based methods, as has been performed for traditional angiography methods [52], is of interest and may improve measurements of contrast agent leakage. We would also like to investigate scanning protocols specifically tailored for leakage imaging to determine the optimal sampling density and interscan time for leakage imaging. Faster imaging could enable more robust characterization, either quantitatively or qualitatively, of the tracer diffusion rates in 3D space. Quantification may also be achieved, for example, by implementing models similar to those used in dynamic light scattering OCT [53] and other autocorrelation-based OCT velocimetry methods [54]. Further studies may also longitudinally characterize the development of leakage in the VLDLR or other disease models to determine the earliest time point that leakage becomes visible with this technique. A formal investigation into the translational potential of ExCEL-OCT is also planned.

## 5. Conclusions

With a single scan, ExCEL-OCT provides structural, angiographic, and leakage information that is separable and both spatially and temporally coregistered. All of this information is provided with high spatial resolution over a full 3D volume. With ExCEL-OCT we demonstrated the first high-resolution volumetric leakage imaging *in vivo.* This new method paves the way for new studies of vascular leakage, and it can be easily implemented in conventional OCT systems across the world.

## Supplemental document

See Supplement 1 for supporting content.
