# Supplementary material for: High-resolution, depth-resolved vascular leakage measurements using contrast-enhanced, correlation-gated optical coherence tomography in mice
Source: Biomed Opt Express. 2021 Mar 2;12(4):1774–91. doi: 10.1364/BOE.415227 (PMC8086440; doi:10.1364/BOE.415227)
Supplement: Supplementary file 2 [file boe-12-4-1774-s001.pdf]

Supplemental Document

Biomedical Optics EXPRESS

High-resolution, depth-resolved vascular leakage measurements using contrast-enhanced, correlation-gated optical coherence tomography in mice: supplement

CONRAD W. MERKLE,[1,*] MARCO AUGUSTIN,[1] DANIELLE J. HARPER,[1] JOHANNA GESPERGER,[1,2] ANTONIA LICHTENEGGER,[1] PABLO EUGUI,[1] GERHARD GARHÖFER,[3] MARTIN GLÖSMANN,[4] AND BERNHARD BAUMANN[1]

[1]*Center for Medical Physics and Biomedical Engineering, Medical University of Vienna, Vienna, Austria*
[2]*Division of Neuropathology and Neurochemistry, Department of Neurology, Medical University of Vienna, Vienna, Austria*
[3]*Department of Clinical Pharmacology, Medical University of Vienna, Vienna, Austria*
[4]*Core Facility for Research and Technology, University of Veterinary Medicine Vienna, Vienna, Austria*
[*]*conrad.merkle@meduniwien.ac.at*

This supplement published with The Optical Society on 2 March 2021 by The Authors under the terms of the Creative Commons Attribution 4.0 License in the format provided by the authors and unedited. Further distribution of this work must maintain attribution to the author(s) and the published article's title, journal citation, and DOI.

Supplement DOI: https://doi.org/10.6084/m9.figshare.13635152

Parent Article DOI: https://doi.org/10.1364/BOE.415227

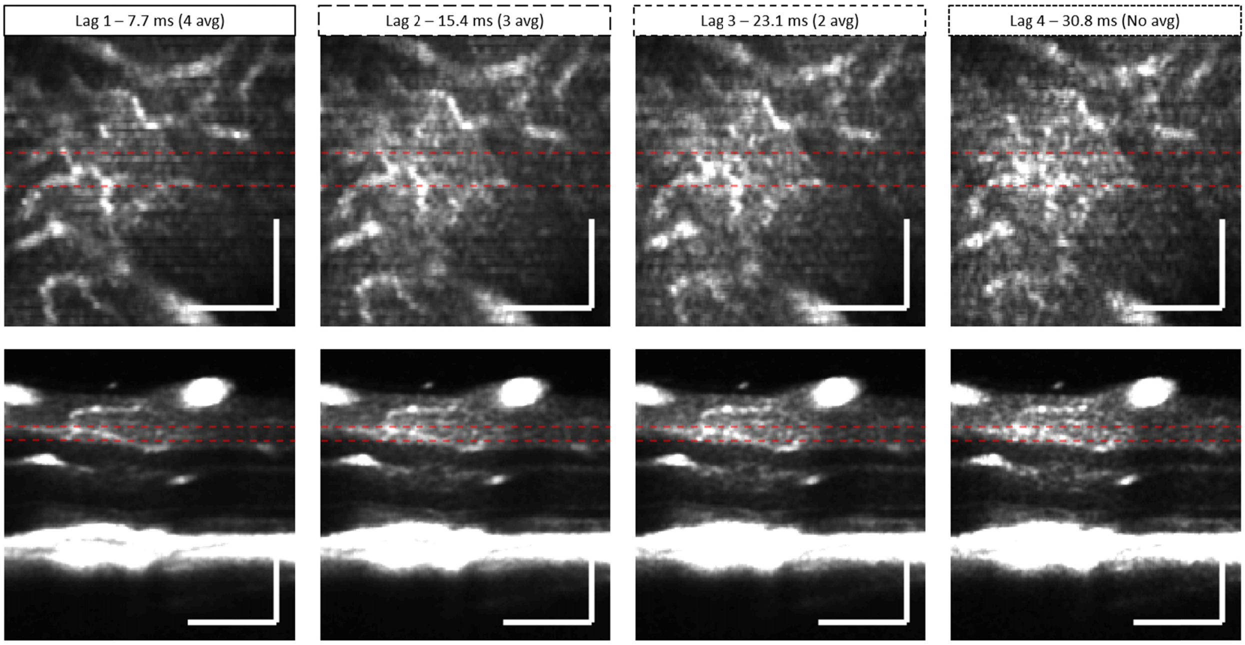

Fig. S1. Zoomed-in view of the leakage region of interest presented in Fig. 2. Mean intensity projections of angiogram signals at different interscan times are shown over 40 µm laterally or 20 µm axially as denoted by the dotted red lines in the enface and B-scan images. Scale bars are 100 µm and all figures are shown with the same dynamic range.

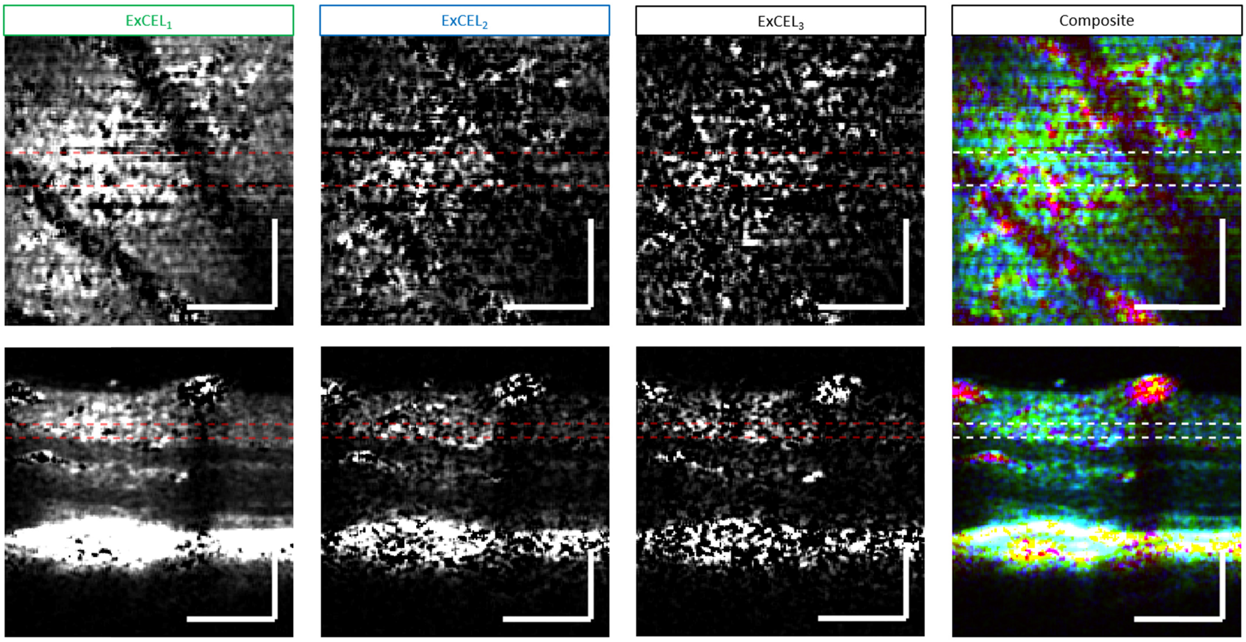

Fig. S2. Zoomed-in view of the leakage region of interest presented in Fig. 3. Mean intensity projections of ExCEL signals with different decorrelation gates are shown over 40 µm laterally or 20 µm axially as denoted by the dotted red or white lines in the enface and B-scan images. A false-color composite image combines the traditional angiogram (red), $ExCEL_1$ (green), and $ExCEL_2$ (blue) signals. Scale bars are 100 µm and all grayscale figures are shown with the same dynamic range.

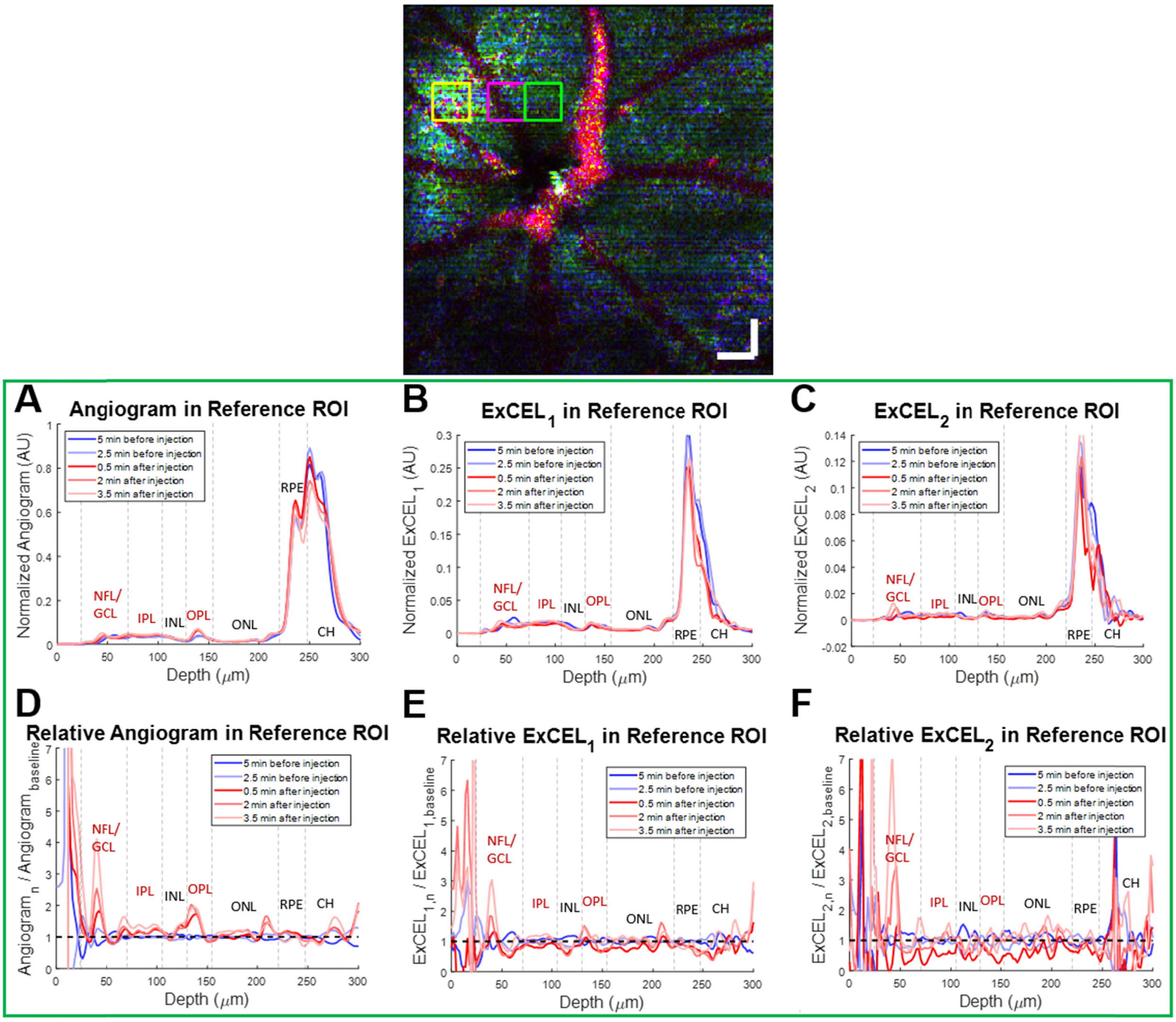

Fig. S3. Depth profiles from a VLDLR mouse eye for the traditional angiogram (A), $ExCEL_1$ (B), and $ExCEL_2$ (C) signals averaged over the 100 µm × 100 µm reference region without apparent leakage or large vascular contributions shown in the false-color image above (green box). Yellow and Magenta boxes mark the positions of depth profiles shown in Figs. 5 and 6 respectively. Relative changes in signals (D-F) were also measured by dividing by the mean baseline profile. Blue profiles were acquired before Intralipid injection and red profiles were acquired after injection. These profiles demonstrate only minor changes in angiogram signal and no apparent changes in ExCEL signals following injection of the lipid tracer. Relative angiogram changes are not as large as those presented in Fig. 6D due to the relatively large ROI area and lower volumetric vascular density. NFL – Nerve Fiber Layer, GCL – Ganglion Cell Layer, IPL – Inner Plexiform Layer, INL – Inner Nuclear Layer, OPL – Outer Plexiform Layer, ONL – Outer Nuclear Layer, RPE – Retinal Pigment Epithelium, CH – choroid. Vascular layers of the inner retina are labeled in red.

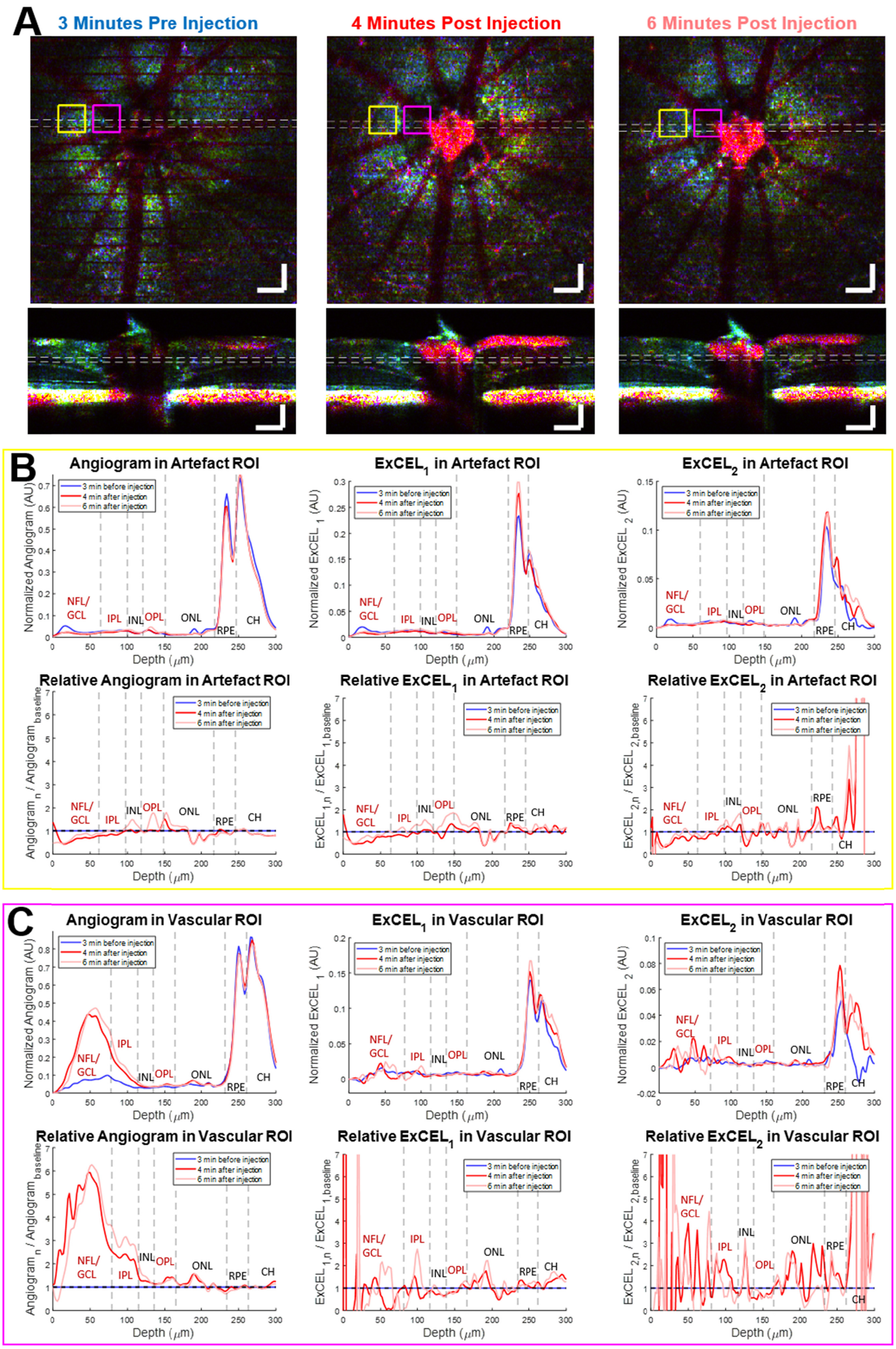

A
3 Minutes Pre Injection
4 Minutes Post Injection
6 Minutes Post Injection
B
Angiogram in Artefact ROI
ExCEL1 in Artefact ROI
ExCEL2 in Artefact ROI
Relative Angiogram in Artefact ROI
Relative ExCEL1 in Artefact ROI
Relative ExCEL2 in Artefact ROI
C
Angiogram in Vascular ROI
ExCEL1 in Vascular ROI
ExCEL2 in Vascular ROI
Relative Angiogram in Vascular ROI
Relative ExCEL1 in Vascular ROI
Relative ExCEL2 in Vascular ROI
3 min before injection
4 min after injection
6 min after injection
NFL/ GCL
IPL
INL
OPL
ONL
RPE
CH
Depth (μm)
Normalized Angiogram (AU)
Normalized ExCEL 1 (AU)
Normalized ExCEL 2 (AU)
Angiogram n / Angiogram baseline
ExCEL 1,n / ExCEL 1,baseline
ExCEL 2,n / ExCEL 2,baseline

Fig. S4. Leakage volume cross sections (A) and depth profiles (B-C) from a wild type mouse eye at multiple time points for the traditional angiogram, $ExCEL_1$, and $ExCEL_2$ signals. Signals were averaged over 100 μm × 100 μm reference regions containing ExCEL signal artefacts (B, yellow box in A) or large vascular contributions (C, magenta box in A). Relative changes in signals were measured by dividing by the pre-injection depth profile. Blue profiles were acquired before Intralipid injection and red profiles were acquired after injection. False coloring of the leakage volume follows the same format as the main text where the angiogram signal is red, and $ExCEL_1$ and $ExCEL_2$ signals are coded green and blue respectively. Leakage imaging (A) shows a ring-shaped artefact surrounding the optic nerve head caused by differences in retinal structure in this region. Following injection of the lipid tracer, the angiogram signal increases within blood vessels, however a difference in the surrounding regions or the ring-shaped artefact is not apparent. The quantitative depth profiles confirm this with no apparent changes in ExCEL signals following injection in either ROI and a large increase in angiogram signal within the vascular ROI (C). Depth profiles in the artefactual region are consistent with those of an ROI in the VLDLR model containing neither leakage nor large vessels (Fig. S3). NFL – Nerve Fiber Layer, GCL – Ganglion Cell Layer, IPL – Inner Plexiform Layer, INL – Inner Nuclear Layer, OPL – Outer Plexiform Layer, ONL – Outer Nuclear Layer, RPE – Retinal Pigment Epithelium, CH – choroid. Vascular layers of the inner retina are labeled in red.
